# Prospective Pathways to Depressive Symptoms and Disordered Eating in Adolescence: **A 7-Year Longitudinal Cohort Study**

**DOI:** 10.1007/s10964-020-01291-1

**Published:** 2020-07-29

**Authors:** Helena Lewis-Smith, Isabelle Bray, Debra Salmon, Amy Slater

**Affiliations:** 1 University of the West of England, Bristol, UK; 2 City University London, London, UK

**Keywords:** Disordered eating, Depression, BMI, Body image, Puberty, Adolescence

## Abstract

Eating pathology and depressive symptoms increase during adolescence, yet predictive pathways remain predominantly unexplored, despite their implications for prevention. The present study aimed to identify shared risk factors for eating pathology and depressive symptoms by evaluating an adapted Dual-Pathway Model of disordered eating, which postulated that higher BMI would predict disordered eating and depressive symptoms via pathways between body dissatisfaction, later BMI, depressive symptoms, and visible indicators of puberty (breast development for girls, height for boys). The participants were 8915 children (49% girls) from the Avon Longitudinal Study of Parents and Children, a population-based cohort study of British children, who were assessed at different intervals between the age of 7 to 14 years. Path analyses revealed that, for girls, childhood BMI exerted indirect effects on disordered eating via body dissatisfaction, depressive symptoms, and more advanced breast development, with indirect pathways identified to depressive symptoms via earlier depressive symptoms and more advanced breast development. For boys, childhood BMI had indirect effects on disordered eating via later BMI and body dissatisfaction, while only earlier depressive symptoms were found to have an independent and direct effect on adolescent depressive symptoms. This study reveals shared and independent risk factors for eating pathology and depressive symptoms in adolescence and suggests targets for preventative interventions, including higher BMI, body dissatisfaction, and depressive symptoms, in addition to advanced breast development, for girls.

## Introduction

Research indicates that eating pathology and depressive symptoms increase during adolescence (Goldschmidt et al. 2016). Both are associated with harmful effects upon physical and psychological health in adulthood, including full threshold eating disorders, anxiety, mood disorders, personality disorders, self-harm, and substance abuse (Bornioli et al. 2019; Johnson et al. 2009). Disordered eating and depressive symptoms often co-occur in adolescence (Goldschmidt et al. 2016), which suggests the likelihood of common underlying causes. Indeed, researchers have called for the examination of how independently studied risk factors interrelate with one another to foster eating pathology and depressive symptoms (Ferreiro et al. 2012), and have recommended targeting shared risk factors for these outcomes in public health interventions (Becker et al. 2014). The Dual-Pathway model of disordered eating (Stice et al. 1996), which includes BMI, body image, and depressive symptoms, in addition to externally visible indicators of puberty, warrant exploration as potential risk factors for these two outcomes. Thus, the present study aimed to identify common childhood predictors, in order to inform the development of early, efficacious and cost-effective interventions to simultaneously prevent depressive symptoms and disordered eating.

The Dual-Pathway model of disordered eating (Stice et al. 1996) postulates shared risk factors for eating pathology and negative affect. Within the model, perceived sociocultural pressure for thinness is argued to promote internalization of appearance ideals, which in turn contributes to body dissatisfaction. It is also hypothesized that a higher body mass index (BMI) will lead to perceived sociocultural pressure for thinness and body dissatisfaction. Finally, it is posited that body dissatisfaction promotes bulimic pathology via the pathways of dietary restraint (restraint pathway) and negative affect/depressive symptoms (negative affect pathway). While originally hypothesizing the development of bulimic symptoms, the Dual-Pathway model and its components have received extensive cross-sectional and prospective support when applied to a series of pathological eating behaviors among adult women and adolescents (e.g., Stice and Van Ryzin 2019). However, only one cross-sectional study has examined the model among children (aged below 10 years; Evans et al. 2013); despite the prevalence of body dissatisfaction (Ricciardelli and McCabe 2001), disordered eating (Erickson and Gerstle 2007), and depressive symptoms (Ghandour et al. 2019), among this younger group. Prospective exploration of the model among pre-adolescents has the potential to inform early interventions to prevent disordered eating and depression in adolescence.

Although the full Dual-Pathway model has not undergone prospective evaluation among children, its central components, including BMI, body dissatisfaction, and depressive symptoms, have received strong empirical support among this young group. First, while the model proposes an indirect/mediated effect of BMI on eating pathology and depressive symptoms via body dissatisfaction, higher childhood BMI has been found to elevate the risk for later body dissatisfaction (Evans et al. 2017), eating pathology (Yilmaz et al. 2019) and depressive symptoms (Evans et al. 2017). Second, body dissatisfaction in childhood has received prospective support as a risk factor for adolescent eating pathology and depressive symptoms (Ferreiro et al. 2014). Third, prospective research indicates that depressive symptoms in childhood and early adolescence increase the risk for subsequent eating pathology (Ferreiro et al. 2012) and depressive symptoms (Heerde et al. 2018).

In addition to these central components of the Dual-Pathway model, puberty has also emerged as a risk factor for depressive symptoms and disordered eating among pre-adolescents. Indeed, adolescent girls at either more advanced stages or earlier onset of pubertal development have been found to experience higher levels of subsequent depressive symptoms (Sequeira et al. 2017) and eating pathology (Baker et al. 2012). In contrast, findings regarding the influence of pubertal timing among boys are less conclusive. While some prospective research suggests that early puberty or later stages of pubertal development indicate a risk factor for depressive symptoms and eating pathology (Baker et al. 2012), other studies suggest the opposite (Conley and Rudolph 2009).

Although there are several different indicators of puberty, they tend to be combined into one composite indicator in research. This practise overlooks the nuances associated with each individual pubertal indicator. For example, some are more externally visible (e.g., breast development, growth spurt) than others (e.g., menarche, body hair growth), and thus may elicit reactions and comments from other people which are likely to have an impact on the individual. Given that breast development, one of the most visible indicators of pubertal development among girls, is often considered to signify femininity and sexuality (Baucom et al. 2005), its psychological impact warrants exploration among adolescents. Similarly, given that height, one of the most visible indicators of puberty among boys, is associated with masculinity (O’Gorman et al. 2019), its psychological effects during adolescence warrant investigation. While breast development and height might contribute to the development of eating pathology and depressive symptoms, their impacts are hypothesized to be different.

In line with the “maturational deviance hypothesis” (Petersen et al. 1980), it is suggested that adolescent girls with more advanced breast development will be more likely to exhibit symptoms of disordered eating and depression. This proposal is based on the argument that girls may not be cognitively prepared for the physical, emotional, and social changes associated with puberty, and may consequently experience adjustment difficulties (Marceau et al. 2011). For example, research suggests that girls with earlier onset of puberty are comparatively more dissatisfied with their body, less popular with their female peers, yet more popular with boys (Compian et al. 2009). This finding may be due to this group having felt unprepared to deal with the objectifying and sexualising attention that their earlier physical development may have evoked from adolescent boys, in addition to experiencing teasing from later-maturing female peers; thus leading to greater self-objectification (Grower et al. 2019). Further, breast development moves girls away from the Westernized appearance ideal for women, which emphasizes a pre-pubertal body shape, and this may increase body dissatisfaction and lead to weight-control behaviors and feelings of depression (Bulik 2002).

In contrast, growing in height is likely to be a more favorable experience for boys, since it moves them closer to the Westernized appearance ideal for men, which emphasizes tall stature among other things (Ricciardelli and McCabe 2004). While body image research has primarily focused on muscularity, qualitative and quantitative studies indicate that height is an important characteristic for men (O’Gorman et al. 2019). In addition to taller stature being associated with greater body satisfaction, it has been related to greater masculinity and dominance (O’Gorman et al. 2019); with shorter males attributing higher levels of dominance to taller men and experiencing lower conformity to masculine norms (Batres et al. 2015). Thus, it has been suggested that shorter men may experience greater difficulty due to internalizing sociocultural attitudes which associate masculine attributes with greater stature (O’Gorman et al. 2019). Growth spurts are one of the most salient indicators of puberty among pre-adolescent boys, and there is greater variance in height among this younger demographic compared with older groups (Haas and Campirano 2006). Further, girls tend to experience growth spurts before their male equivalents (Haas and Campirano 2006). Finally, taller boys and those who experience an earlier onset of puberty have been deemed more popular and attractive with girls compared with their later developing peers (Cawley et al. 2006). Collectively, this suggests that adolescent boys who are taller are likely to have a more favorable experience, while shorter boys will be at greater risk of experiencing later eating pathology and depressive symptoms.

It is important to note that in addition to the child-specific biological and psychological factors under study in the present research, there are many other risk factors for disordered eating and depressive symptoms, including maternal influences. For example, both maternal education and social class have been associated with eating pathology and depression risk (e.g., Ahrén-Moonga et al. 2009), as well as maternal age at birth of the child (e.g., Tearne et al. 2016). Further, it is unsurprising that studies have found maternal history of an eating disorder to be a risk factor for eating disorders (Field et al. 2008) and depression (Cimino et al. 2015). Similarly, maternal history of depression has also been found to predict the child’s development of disordered eating (Cimino et al. 2015) and depression (Pearson et al. 2013).

While most prospective studies exploring risk factors for eating pathology and depression have focused on, and operationalised, these outcomes as full-threshold classifications (e.g., Anorexia Nervosa, Major Depressive Disorder, as per the Diagnostic and Statistical Manual of Mental Disorders, Fifth [DSM-V]; Stice and Van Ryzin 2019), fewer have focused on the presence of individual symptoms associated with these classifications (e.g., fasting for Anorexia Nervosa, feelings of hopelessness for Major Depressive Disorder), which are more commonly experienced in the general population. Adoption of a more inclusive and broader approach which focuses on the presence of these symptoms will produce findings with implications for the wider population, as distinct from the subgroup with full-threshold disorders.

## Current Study

**Fig. 1:**
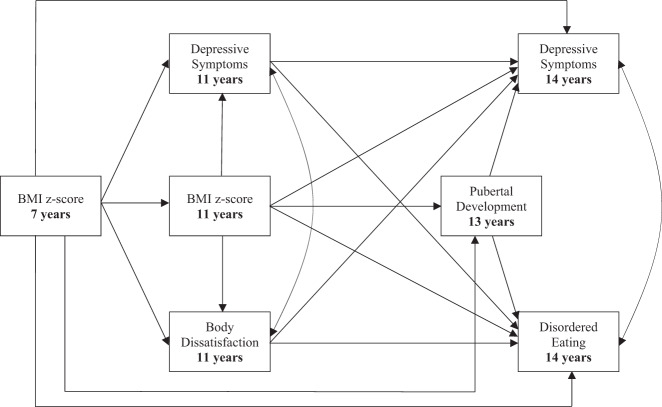
Hypothesized model predicting depressive symptoms and disordered eating in adolescence

Research indicates the increase of eating pathology and depressive symptoms throughout adolescence. They are associated with negative impacts upon physical and psychological health, and thus highlight the importance of identifying risk factors to inform preventive efforts. Both the Dual-Pathway model of disordered eating and pubertal development have received empirical support for their contribution to the development of depression and eating pathology. However, previous studies have had a number of shortcomings. First, they have focused on adolescents, without exploring these risk factors among younger pre-adolescents. Second, they have tended to group all indicators of puberty together, without considering the individual impacts of each indicator. Third, they have mostly considered outcomes of eating pathology and depression as full-threshold classifications, while overlooking the associated individual symptoms. Therefore, the present study aimed to prospectively identify common risk factors in pre-adolescence for disordered eating and depressive symptoms in adolescence, by evaluating an adaptation of the Dual-Pathway model of disordered eating, which incorporated the impact of external indicators of puberty. This was examined separately among genders, and across seven years, spanning childhood to early adolescence. In line with the adapted model, the first hypothesis was that among both genders, higher childhood BMI would predict adolescent disordered eating and depressive symptoms via pre-adolescent body dissatisfaction, depressive symptoms, and higher BMI (see Fig. 1). The second hypothesis was that among girls, more advanced breast development would predict disordered eating and depressive symptoms, as they would feel cognitively unprepared for the associated reception of sexualised attention and the move away from the Westernized appearance ideal for women. The third hypothesis was that among boys, shorter stature would predict disordered eating and depressive symptoms, due to the association of taller stature with masculinity and the Westernized appearance ideal for men. There were several covariates, including maternal socioeconomic status (as indicated by maternal education and social class), maternal age at birth of the child, maternal history of anorexia and bulimia, and maternal history of depression.

## Methods

### Participants

Participants were enrolled in the Avon Longitudinal Study of Parents and Children (ALSPAC), a population-based study of women and their children (Boyd et al. 2013; Fraser et al. 2013; Golding et al. 2001). All pregnant women residing in the Avon area of the UK, with an estimated date of delivery between 01/04/1991 and 31/12/1992, were invited to participate. The initial cohort included 14,531 pregnancies, with 13,988 children alive at 1 year (52% males and 48% females). At 7 years of age, an additional 713 children were enrolled into the study (phases 2 and 3). Informed written consent was obtained from the mothers, while children also provided their consent at later timepoints. Ethical approval for the study was obtained from the ALSPAC Ethics and Law Committee and the Local Research Ethics Committees. A full list of the research ethics committee is available at: http://www.bristol.ac.uk/alspac/researchers/research- ethics/. Details about ALSPAC, including a fully searchable data dictionary, are available on the study website: www.bris.ac.uk/alspac.

For the present study, all children who had a clinic measure of BMI at age 7 were included in these analyses (*N* = 8195; 49% girls; 51% boys), and full information maximum likelihood (FIML) was used to address missing data in the path analyses. Most of the present sample were of white ethnicity (85.8.%) and were born to a mother aged between 25 and 34 years of age (68.2%). With regard to maternal education, more than a third had obtained either A-levels or a university degree (38.7%). With regard to maternal occupation, most were in non-manual jobs (64.1%). Collectively, this suggests the participants were of medium-high socioeconomic background. Table 1 provides further sociodemographic information for the entire sample as well as information by gender.

**Table 1:** Sociodemographic and covariate characteristics for the full sample and by gender

Characteristic	Number of respondents	Full sample (*N* = 8195) *n* (%)	Girls (*n* = 4040) *n* (%)	Boys (*n* = 4155) *n* (%)
Child ethnicity	7333			
White		7035 (85.8)	3454 (85.5)	3581 (86,2)
Non-white		298 (8.6)	152 (3.8)	146 (3.5)
Maternal education	7448			
University degree		1199 (14.6)	609 (15.1)	590 (14.2)
A-level		1977 (24.1)	993 (24.6)	984 (23.7)
GCSE or O-level		3363 (41.0)	1614 (40.0)	1749 (42.1)
Vocational		647 (7.9)	306 (7.6)	341 (8.2)
Certified secondary education		262 (3.2)	134 (3.3)	128 (3.1)
Maternal social class	6671			
Professional		275 (3.4)	130 (3.2)	145 (3.5)
Managerial and technical		2134 (26.0)	1050 (26.0)	1084 (26.1)
Skilled non-manual		2847 (34.7)	1390 (34.4)	1457 (35.1)
Skilled manual		237 (2.9)	113 (2.8)	124 (3.0)
Partly skilled		990 (12.1)	468 (11.6)	522 (12.6)
Unskilled		188 (2.3)	92 (2.3)	96 (2.3)
Maternal age at delivery	7746			
<25 years		1214 (14.8)	617 (15.2)	597 (14.4)
25-29 years		3061 (37.4)	1507 (37.3)	1554 (37.4)
30-34 years		2524 (30.8)	1254 (31.0)	1270 (30.6)
35+		947 (11.6)	429 (10.6)	518 (12.5)
Maternal history of an eating disorder	6412	278 (3.4)	135 (3.3)	143 (3.4)
Anorexia nervosa		138 (1.7)	61 (1.5)	77 (1.9)
Bulimia		201 (2.5)	104 (2.6)	97 (2.3)
Maternal history of depression	5737	1258 (15.4)	639 (15.8)	619 (14.9)

*Note:* Calculations based on data available, hence percentages will not add up to 100%

### Measures

All participants completed measures either via postal questionnaires or by computer at a clinic visit. Anthropometric measurements were taken by nurses at the clinic. Additional details and descriptive statistics regarding the measures are reported in Table 2.

**Table 2:** Descriptive statistics and correlations amongst variables for boys and girls

Girls												
Variable	Number of respondents	Source	Scale range	Mean	SD	Variable						
						1	2	3	4	5	6	7
1. BMI z-score (7 years)	4040	Clinic	–	0.14	0.03							
2. BMI z-score (11 years)	3287	Clinic	–	0.25	1.17	0.87**						
3. Depressive symptoms (11 years)	3253	Clinic	0 – 26	3.84	3.49	0.01	0.26					
4. Body dissatisfaction (11 years)	3038	Questionnaire	−4 – +4	0.26	0.68	0.50**	0.57**	0.06*				
5. Breast developmental stage (13 years)	2500	Questionnaire	1 – 5	3.5	0.90	0.26**	0.30**	−0.02	0.13**			
6. Depressive symptoms (14 years)	2735	Clinic	0 – 26	5.73	4.91	0.07**	0.08**	0.26**	0.07**	0.10**		
7. Disordered eating (14 years)	2588	Questionnaire	0 – 8	1.63	1.58	0.34**	0.37**	0.18**	0.27**	0.16**	0.35**	

Boys												
Variable	Number of respondents	Source	Scale range	Mean	SD	Variable						
						1	2	3	4	5	6	7
1. BMI z-score (7 years)	4155	Clinic	–	0.13	1.06							
2. BMI z-score (11 years)	3249	Clinic	–	0.36	1.16	0.86**						
3. Depressive symptoms (11 years)	3196	Clinic	0 – 26	4.14	3.44	0.00	0.01					
4. Body dissatisfaction (11 years)	2791	Questionnaire	−4 – +4	0.07	0.64	0.48**	0.53**	0.04*				
5. Height z-score (13 years)	2926	Clinic	–	0.45	1.05	0.26**	0.30**	−0.03	0.13**			
6. Depressive symptoms (14 years)	2636	Clinic	0 – 26	4.13	3.85	−0.00	0.02	0.33**	0.04	0.01		
7. Disordered eating (14 years)	2036	Questionnaire	0 – 8	0.73	1.11	0.34**	0.40**	0.11**	0.25**	0.08**	0.16**	

*p < 0.05; **p < 0.001

#### BMI

BMI was calculated using height and weight, which were measured at a mean age of 7.42 years (hereafter referred to as 7 years) and 10.67 years (hereafter referred to as 11 years). The zanthro function in Stata was used to obtain BMI z-scores, which were adjusted for gender and age using UK references (e.g., Cole et al. 2007).

#### Body dissatisfaction

Body dissatisfaction was assessed at a mean age of 10.75 years (hereafter referred to as 11 years) using the Body Image Perception and Attitude Scale for Children (BIPAS-C; Dowdney et al. 1995). The BIPAS-C comprises a five-figure rating scale and children are required to select both the figure they perceive to be most similar to their own and their “ideal” figure. A score (range -4 to +4) is derived by subtracting the ideal figure from the perceived figure. Scores other than zero reflect body dissatisfaction; negative values indicate a desire to be larger, positive values indicate a desire to be thinner. This tool has demonstrated good test-retest reliability and validity (Dowdney et al. 1995).

#### Depressive symptoms

Depressive symptoms were assessed at a mean age of 10.67 years (hereafter referred to as 11 years) and 13.80 years (hereafter referred to as 14 years) using the Short Moods and Feelings Questionnaire (SMFQ; Messer et al. 1995). The SMFQ is a 13-item questionnaire which assesses depressive symptoms over the previous two weeks with higher scores indicating more severe depressive symptoms (range 0-26). The SMFQ has a high correlation with clinical measures of depression (Turner et al. 2014) and has indicated good internal reliability among similar ALSPAC samples previously (e.g., Kwong et al. 2019; Cronbach *a* = 0.80–0.87).

#### Pubertal development

At a mean age of 13.17 years (hereafter referred to as 13 years), girls completed the Tanner staging for breast development (Marshall and Tanner 1969), whereby they selected one of five line drawings (and descriptions) which they felt most closely matched their stage of breast development (Morris and Udry 1980). Since the ordered categorical variable indicated a linear relationship between breast developmental stage and the two outcomes of interest, the variable was treated as continuous, as per previous studies (e.g., Joinson et al. 2012). At a mean age of 12.83 years (hereafter referred to as 13 years) boys had their height measured, with Z-scores obtained using the zanthro function in Stata (Cole et al. 1998).

#### Disordered eating

Disordered eating was assessed at a mean age of 14.08 years (hereafter referred to as 14 years) using eight questions adapted from the Youth Risk Behavior Surveillance System questionnaire (YRBSSQ; Kann et al. 1996). The YRBSSQ has undergone validation among adolescents (Field et al. 2004) and indicates good test-retest reliability (Brener et al. 1995). The current study used eight questions relating to symptoms of disordered eating to create a disordered eating scale. The first of these questions examined the respondent’s current efforts in relation to their body weight (i.e., whether they were attempting to change their weight). The four categorical responses included: I am not trying to do anything about my weight; I am trying to gain weight; I am trying to stay the same; I am trying to lose weight. The first three responses were collapsed to create a binary variable indicating current efforts to lose weight versus no current efforts for weight loss. The next five questions assessed the frequency of engagement in particular behaviors with the intention of losing weight over the previous year. The five behaviors were dieting, exercising, fasting, purging, and laxative use. Each question had 5-6 categorical responses reflecting different frequencies e.g., Never; Less than once a month; 1-3 times a month; Once a week; 2 or more times a week. For all five questions, all responses indicating any engagement with the behaviors (regardless of the frequency) were collapsed to create a binary variable indicating any engagement in the particular behavior with the aim of losing weight versus no engagement. The seventh question assessed the frequency of engaging in an “eating binge”, described as eating an amount of food that most people would consider to be very large, in a short period of time. There were five categorical responses (Never; Less than once a month; 1–3 times a month; Once a week; More than once a week), which were collapsed to create a binary variable indicating any frequency of engagement in an “eating binge” versus no engagement. The eighth and final question asked whether the respondent had ever been suspected by others of having an eating disorder. The four responses were: No; Yes, a friend; Yes, a parent; Yes, a doctor, nurse, or other health care provider. The responses were collapsed to create a binary variable indicating whether the individual had ever been suspected of having an eating disorder versus not.

Collapsing the first seven questions into binary variables allowed an indication of whether the individual exhibited that particular symptom of disordered eating (YES) or not (NO). The final question also served as a further indication of the individual exhibiting eating disorder symptoms, by indicating whether they had been suspected by someone of having an eating disorder (YES) or not (NO). Given that the focus of the present research was to examine symptoms of disordered eating among the general population (as distinct from examining threshold eating disorders), the responses for the eight questions were summated to create an ordinal total disordered eating scale. Therefore, the total score could range between 0 (zero symptoms) and 8 (eight symptoms). This method of creating an ordinal score of disordered eating has been used previously (Bornioli et al. 2019).

#### Covariates

Various covariates suggested by the literature as possible confounders were included in the present analyses. Means and standard deviations for these are reported in Table 1.

##### Maternal education

Maternal highest educational qualification was classified using five categories (Certified Secondary Education, Vocational, GCSE or O level, A level, University degree). This was self-reported at 32 weeks gestation.

##### Maternal social class

Maternal social class was determined by occupation, which was classified using six categories (Unskilled, Partly skilled, Skilled manual, Skilled non-manual, Managerial and technical, Professional). This was self-reported at 32 weeks gestation.

##### Maternal age at childbirth

Maternal age at birth of the child was derived from self-report questionnaires.

##### Maternal history of an eating disorder

Mothers were asked whether they had ever had anorexia or bulimia in the past when children were at a mean age of 8.20 years. The three categorical responses included: Yes, had it recently (in the past year); Yes, in past, not recently; No, never. The first two responses were collapsed, leading to a binary variable indicating whether the individual had any history of anorexia or bulimia versus none.

##### Maternal history of depression

Mothers were asked whether they had had depression in the previous two years when children were at a mean age of 12.16 years. The three categorical responses included: Yes, and consulted doctor; Yes, but did not consult doctor; No. The first two categories were collapsed to create a binary variable indicating whether the individual had experienced depression.

### Attrition

Based on the sample of participants for whom a clinic measure of BMI at 7 years of age was available (*n* = 8,195), levels of missingness ranged between 5% and 44%. The number of respondents for each respective measure in the present study are indicated in Table 2. With regard to attrition, response rates to the questionnaires for the timepoints examined in the present study ranged between 57-67% (for more information regarding ALSPAC attrition, see Boyd et al. 2013), and are comparable to other cohort-based studies (Copeland et al. 2011). Missing data patterns in the present study were assessed, and as is typically the case for family cohort studies, the data were indicated as missing at random (MAR; Acock 2005). Variables predicting attrition included lower maternal social class, maternal history of depression, child’s BMI and depressive symptoms at 11 years of age. These variables were used to estimate missing data by FIML estimation, and the maternal variables were included as covariates. This approach was adopted to reduce bias, maximize the use of all available data, and maximize recoverability of “true” scores (Little and Rubin 2014).

### Statistical Analysis

Analyses were conducted to test the hypotheses for boys and girls separately. Bivariate correlations between the model variables were explored and informed the hypothesized model (Fig. 1). This model was tested separately among each gender using path analysis, a structural equation modeling (SEM) technique. All potential confounders were included as covariates. Model fit was assessed using Chi-square, the Root Mean Square of Approximation (RMSEA), and the Comparative Fit Index (CFI). A RMSEA value of 0 indicates perfect fit, while a CFI value closer to 1 indicates better fit. Bias-corrected bootstrap analyses were conducted to test the statistical significance of direct and indirect effects between BMI z-scores at 7 years and both disordered eating and depressive symptoms at 14 years of age. Descriptive statistics and correlational analyses were conducted in Stata 14 (StataCorp. 2015). Path analyses were carried out in Mplus V7.4 (Muthen and Muthen 1998).

## Results

### Descriptive and Correlational Analysis

The means, standard deviations, and scale ranges for model variables are included in Table 2. This also includes the inter-correlations between the model variables.

For girls, there were no significant correlations between BMI z-scores at ages 7 and 11 and depressive symptoms at 11 years. There was also no significant correlation between depressive symptoms at age 11 and breast developmental stage at 13 years. However, there was strong evidence of correlations between all other variables. In both genders, most of the correlations revealed patterns of association that were consistent with the hypothesized model. For boys, there were no significant correlations between BMI z-scores at ages 7 or 11 and depressive symptoms at 11 or 14 years. However, BMI z-scores at both 7 and 11 indicated significant positive correlations with body dissatisfaction at 11 years, height z-scores at 13 years and disordered eating at 14 years. While depressive symptoms at 11 years was the only variable showing a significant relationship with depressive symptoms at 14 years, all variables showed significant positive correlations with disordered eating at 14 years.

### Path Analyses

**Fig. 2:**
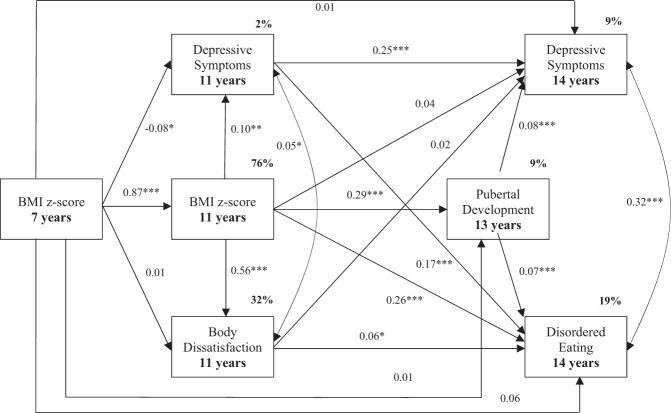
Path standardized coefficients and percentage of variance explained for the model for girls. *p < 0.05; **p < 0.01; ***p < 0.001

Among girls, the hypothesized model revealed a very good fit to the data (*χ*^2^ = 8.918, *p* = 0.01; RMSEA = 0.03; CFI = 1.00). Figure 2 illustrates the standardized path coefficients for the model and indicates significant pathways. With regard to the first hypothesis, BMI z-scores at 7 years exerted significant total effects on depressive symptoms at 14 years of age (β = 0.07, *SE* = 0.02, *p* = 0.00), but these effects were completely mediated by other variables. Indirect effects were found via the pathway from BMI at 11 years to depressive symptoms at age 11 (β = -0.02, *SE* = 0.01, *p* = 0.01), and via the direct pathway from depressive symptoms at 11 years (β = -0.02, *SE* = 0.01, *p* = 0.04). Similarly, BMI z-scores at 7 years exerted significant total effects on disordered eating at 14 years (β = 0.34, *SE* = 0.02, *p* = 0.00), yet these were mediated via various pathways. Indirect effects were identified via the direct pathways from depressive symptoms at 11 years (β = -0.01, *SE* = 0.01, *p* = 0.04) and from BMI at 11 years (β = 0.23, *SE* = 0.04, *p* = 0.00) to disordered eating at 14 years. However, there were further indirect effects via the subsequent pathways from BMI at 11 years to depressive symptoms at 11 years (β = 0.01, *SE* = 0.01, *p* = 0.01) and body dissatisfaction at 11 years (β = 0.03, *SE* = 0.01, *p* = 0.01). With regard to the second hypothesis, indirect pathways were identified from BMI at 7 and 11 years to depressive symptoms (β = 0.02, *SE* = 0.01, *p* = 0.00) and disordered eating (β = 0.02, *SE* = 0.00, *p* = 0.00) via breast development stage at 13 years. The model explained 19% of the variance in disordered eating, 9% depressive symptoms (14 years), 9% in breast developmental stage, 32% in body dissatisfaction, 2% in depressive symptoms (11 years), and 76% of the variance in BMI (11 years).

**Fig. 3:**
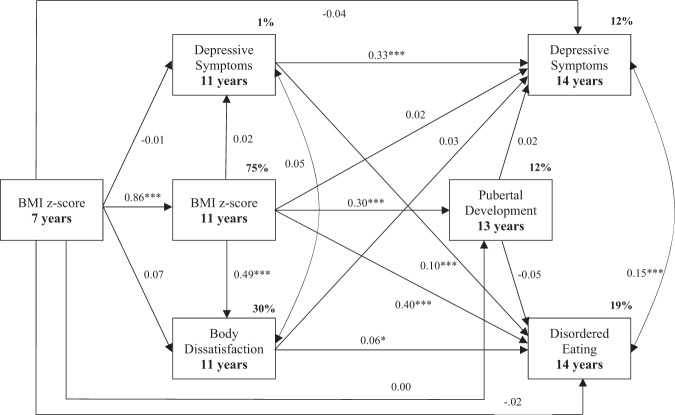
Path standardized coefficients and percentage of variance explained for the model for boys. *p < 0.05; **p < 0.01; ***p < 0.001

Among boys, the hypothesized model revealed an excellent fit to the data (χ^2^ = 3.698, *p* = 0.16; RMSEA = 0.01; CFI = 1.00). Figure 3 illustrates the standardized path coefficients and indicates significant pathways. With regard to the first hypothesis, BMI z-scores at 7 years exerted significant total effects on disordered eating at 14 years (β = 0.36, *SE* = 0.04, *p* = 0.00). In addition to exerting effects via BMI at age 11 (β = 0.34, *SE* = 0.04, *p* = 0.00), marginally significant indirect effects were identified via the pathway from BMI at 11 years to body dissatisfaction at 11 years (β = 0.03, *SE* = 0.01, *p* = 0.05). Depressive symptoms at 11 years did not carry indirect effects, but instead exerted direct effects on disordered eating and depressive symptoms (14 years). With regard to the third hypothesis, an indirect effect of height at 13 years (via BMI at 7 and 11 years) on disordered eating at 14 years approached significance (β = -0.01, *SE* = 0.01, *p* = 0.08), while this was non-significant in relation to depressive symptoms (β = 0.02, *SE* = 0.02, *p* = 0.44). The model explained 19% of the variance in disordered eating, 12% in depressive symptoms (14 years), 11% in height, 30% in body dissatisfaction, 1% in depressive symptoms (11 years), and 75% of the variance in BMI (11 years).

## Discussion

Research indicates a co-occurrence and increase in disordered eating and depressive symptoms throughout adolescence (Goldschmidt et al. 2016), both of which predict negative impacts on health in adulthood (Bornioli et al. 2019; Johnson et al. 2009). This highlights the importance of early prevention, which could be aided through the identification of common risk factors. The Dual-Pathway model proposes pathways to the development of eating pathology and depressive symptoms via BMI, perceived sociocultural pressure for thinness, and body dissatisfaction (Stice et al. 1996). Central components of the model have received individual empirical support for the prediction of eating pathology and depressive symptoms among pre-adolescents (e.g., Evans et al. 2017). However, the interrelated pathways have not undergone prospective exploration among this younger group. Puberty has also emerged as a risk factor for eating pathology and depressive symptoms (Lewis et al. 2018), yet research has neglected the individual impact of externally visible pubertal indicators, including breast development for girls and height for boys, which may elicit social reactions that have an adverse impact on the individual. The present study therefore aimed to fill these gaps in knowledge by examining risk factors for, and interrelated pathways to, eating pathology and depressive symptoms in adolescence. This was done by evaluating an adaptation of the Dual-Pathway model of disordered eating across seven years, spanning childhood to early adolescence (see Fig. 1).

With regard to the first hypothesis, findings differed by gender. For girls, early BMI was found to predict disordered eating and depressive symptoms in adolescence via different pathways in the model. With regard to the outcome of disordered eating, effects of childhood BMI were mediated by pre-adolescent BMI, depressive symptoms, and body dissatisfaction. The mediating influence of body dissatisfaction supports previous findings (Jendrzyca and Warschburger 2016) and indicates that girls of higher adiposity are vulnerable to engaging in disordered eating due to dissatisfaction with their bodies. However, the differing impact of BMI at ages 7 and 11 on pre-adolescent depressive symptoms was unexpected. This is the first study to reveal adverse impacts associated with lower childhood BMI on subsequent depressive symptoms and contradicts previous prospective research conducted among this group (Evans et al. 2017). There may be several different reasons for these inconsistent findings. One may be the differing use of measures. For example, Evans et al. (2017) employed the short form of the Children’s Depression Inventory (CDI-S; Kovacs 1992), which unlike the SMFQ employed in the present study, includes an item related to appearance: (“I look ugly”). Children of a higher BMI have been found to experience greater weight stigma (Jendrzyca and Warschburger 2016), and thus may deem themselves less attractive, which may give rise to greater leading them to indicate a higher degree of agreement with this item on the CDI-S. An alternative consideration is that girls as young as 7 may be less aware of, or vulnerable to, societal standards for thinness. Instead, they may value heavier bodies, as they have been associated with muscle and stature relating to sporting success (Tatangelo and Ricciardelli 2013). Finally, this unexpected finding could be an anomaly, and BMI at such an early age may not be associated with depressive symptoms. Indeed, a prospective study which examined this association at regular intervals between the ages of 2 and 12 found BMI to be influential from 8 years of age, with the authors suggesting that the social-cognitive processes responsible for forging this connection (e.g., experiencing weight stigma, body dissatisfaction) may not undergo development until this age (Bradley et al. 2008). Nonetheless, these are all speculations and further examination of this unexpected finding is needed. The subsequent impact of depressive symptoms on disordered eating mirrors previous findings (Gardner et al. 2000), and suggests that girls who engage in disordered eating may do so due to negative affect, whereby they engage in “comfort” eating to cope with their feelings rather than trying to alter their body weight and shape (Stice et al. 1996). Finally, the direct pathway from pre-adolescent BMI to disordered eating supports previous findings (Reed et al. 2017). With regard to the outcome of depressive symptoms in adolescence, the first two identified pathways via pre-adolescent depressive symptoms and BMI at 11 years have already been discussed. Contrary to predictions, body dissatisfaction was not found to predict later depressive symptoms. This suggests that while girls of higher adiposity may experience negative effect, this may not be due to feelings of body dissatisfaction. This emphasizes that the direct effects of BMI on both disordered eating and depressive symptoms warrant further exploration, as other variables not currently accounted for in the model may be acting as mediators, including internalization of appearance ideals (Evans et al. 2013), weight teasing/stigma (Vartanian and Porter 2016), and “body talk” (conversations about appearance which reinforce appearance pressures; Strandbu and Kvalem 2014).

With regard to the first hypothesis in relation to the boys, there was greater support for the pathways leading to eating pathology within the model. The identification of the first impact of childhood BMI via pre-adolescent BMI, and subsequently body dissatisfaction, supports previous research which found that boys with higher adiposity were more likely to develop disordered eating due to dissatisfaction with their body (Jendrzyca and Warschburger 2016). The second direct pathway from pre-adolescent BMI to disordered eating mirrors previous findings (e.g., Reed et al. 2017) and simultaneously suggests that BMI may exert additional effects via other mediators. One potential mediator may be weight stigma (including weight-related teasing). Cross-sectional research has consistently identified an association between the experience of weight stigma and disordered eating (Vartanian and Porter 2016), with prospective research also having identified weight teasing as a predictor among boys (Haines et al. 2006). Therefore, it could be argued that boys of higher adiposity experience teasing about their weight, and therefore engage in restrictive eating to avoid future experiences of weight stigma and teasing. Finally, the independent direct effect of pre-adolescent depressive symptoms on disordered eating may be unrelated to BMI or body dissatisfaction. This previously observed effect (Gardner et al. 2000) may be due to boys engaging in disordered eating for self-soothing of depressive symptoms and not in an attempt to control their weight. Contrary to predictions, only the prevalence of pre-adolescent depressive symptoms emerged as a risk factor for depressive symptoms in adolescence. This risk factor has been identified previously (Heerde et al. 2018) and highlights the persistent nature of negative affect among boys. The absence of evidence connecting BMI with depressive symptoms supports previous findings (Evans et al. 2017), which indicates that the association between adiposity and negative affect tends to emerge later in childhood among boys. Similarly, the present findings suggest that body dissatisfaction does not foster depressive symptoms, and thus replicates previous studies (Paxton et al. 2006) while challenging others (Goldschmidt et al. 2016). However, research has also indicated that body dissatisfaction becomes a salient risk factor for depressive symptoms later in adolescence (Paxton et al. 2006). Collectively, this implies that BMI and body dissatisfaction may be less influential in the development of depressive symptoms among boys in earlier adolescence. The conflicting findings in the literature also highlight the importance of developing measures to capture male-specific body image concerns. Many traditional measures, including the figure rating scale utilized in the present study (Dowdney et al. 1995), tend to focus on weight and shape concerns, and are thus unlikely to be capturing male concerns, such as muscularity (Ricciardelli and McCabe 2004). Nonetheless, there are likely to be other aspects of boys’ self-esteem which might influence and contribute towards depressive symptoms, such as perceived sports ability (Slutzky and Simpkins 2009).

The second hypothesis regarding the adverse impact of more advanced breast development on the development of eating pathology and depressive symptoms among girls was supported. This study is also the first to examine breast development as a risk factor for disordered eating, and advances prior research which found earlier puberty, measured via other indicators, to predict disordered eating (Baker et al. 2012). The present findings may be attributable to girls with more advanced breast development feeling unprepared for the emotional and social changes associated with their visible indicator of maturity (e.g., Ge et al. 2003). This group may feel uncomfortable with the associated objectifying and sexualising attention they experience from adolescent boys, while also experiencing teasing from later-developing peers (Grower et al. 2019). This may lead them to engage in disordered eating in an attempt to “lose” their breasts, and thus receive less attention. An alternative explanation may be that adolescent girls with advanced breast development are being pushed away from the Westernized appearance ideal for women, and thus pursue weight-control behaviors in an attempt to change their bodies (Bulik 2002). These postulations may also relate to the observed adverse impact of advanced breast development on the development of depressive symptoms, which mirrors previous findings (e.g., Joinson et al. 2012). However, these potential mediating mechanisms are all speculative and warrant further exploration.

The third hypothesis regarding the adverse impact of shorter stature on the development of eating pathology and depressive symptoms among boys was not supported. While the effects of childhood and pre-adolescent BMI on height strengthen previous cross-sectional findings (Bosy-Westphal et al. 2009), the absence of subsequent effects on depressive symptoms contradicts other cross-sectional research (Rees et al. 2009). However, the latter study found that among adolescents aged 12–19 years, an association between height and depressive symptoms only emerged among boys aged 14 and above (Rees et al. 2009), which suggests that height may become more influential on depressive symptoms later in adolescence. In contrast, the impact of shorter stature on disordered eating approached significance (*p* = 0.08), which lends partial support to previous prospective findings (Gardner et al. 2000). As a Western appearance ideal for men (Fornari and Dancyger 2003), it is not surprising that height has been found to be an important source of appearance satisfaction among adult men (Griffiths et al. 2019). In view of this, the present findings raise the possibility that boys of shorter height may experience dissatisfaction with this uncontrollable aspect of their appearance, which consequently leads them to engage in compensatory disordered eating behaviors in an attempt to change other aspects of their appearance; for example, to attain a slim and muscular body shape. However, this supposition warrants further examination, in addition to previously indicated moderating factors, such as conformity to masculine norms (O’Gorman et al. 2019). As may be the case with depressive symptoms, it is possible that height becomes increasingly influential on disordered eating later in adolescence. Indeed, increasing numbers of boys engage in romantic relationships the further they move through adolescence (Carver et al. 2003), and research indicates that those who are taller or at later stages of puberty are deemed more popular, attractive, and are more likely to be dating compared to their shorter peers (Cawley et al. 2006). Thus, there is likely to be increasing pressure relating to height as boys move through adolescence. This warrants further exploration and highlights the importance of assessing multiple aspects associated with male appearance ideals, such as muscularity and height.

While the present findings have important implications, the limitations warrant consideration. First, the sample lacks socio-economic and ethnic diversity, indicating that the findings may be less generalizable. Second, the measure of disordered eating was not a validated tool, and instead was based on a series of questions relating to eating behaviors that were available for the cohort. However, the questions were based on those of a validated instrument (e.g., Youth Risk Behavior Surveillance System questionnaire; Kann et al. 2000). Third, while a simple cross-sectional measure of one indicator of puberty was employed, the difficulty of identifying truly unique effects of a particular pubertal indicator should be acknowledged, given that developmental maturation across the different indicators tends to be highly correlated (Schubert et al. 2005). Fourth, although this is a longitudinal study, three of the key variables (BMI, body image, and depression) were measured at approximately the same age (11 years). Therefore, while strong evidence of associations between these variables has been found, it is not possible to draw conclusions about the direction of causation. Finally, the data was collected in the 1990s and 2000s, and thus does not account for the more recent impact of social media, which has been found to predict disordered eating and depressive symptoms (Primack et al. 2017). It is therefore recommended that future research replicates the present examination in more recent cohorts, while accounting for this additional influence.

Nonetheless, this study has a number of strengths. First, it has tested an adaptation of a well-supported theory, the Dual-Pathway of disordered eating (Stice et al. 1996), and has identified novel etiological pathways to the development of eating pathology and depressive symptoms among adolescents, including the impact of breast development stage among girls. Second, the present study has focused on symptoms of disordered eating and depressive symptoms, as distinct from their clinical threshold classifications. This has enabled a broader exploration of etiological pathways to disordered eating and depressive symptoms at a population level and has important population-based implications. Given the risk factors of BMI, body dissatisfaction, and external indicators of puberty; the implementation of early interventions which target these variables is indicated. However, instead of implementing programmes promoting weight loss among children, school-based interventions which foster positive body image and promote the acceptance of diverse bodies and appearance are recommended (Bray et al. 2018). Such an approach would also help prepare preadolescents for puberty and it associated bodily changes.

## Conclusion

Symptoms of eating pathology and depression have been found to increase during adolescence. Both are associated with adverse consequences on physical and psychological health in adulthood, thus warranting concern. Their co-occurrence during adolescence suggests the possibility of common underlying causes, which, if targeted, in interventions, might facilitate simultaneous prevention. The present longitudinal study therefore sought to identify common childhood predictors of eating pathology and depressive symptoms by evaluating an adapted version of the Dual-Pathway model of disordered eating (Stice et al. 1996) which incorporated the influence of externally visible indicators of pubertal development. Findings revealed partial support for the model among both genders. Among girls, BMI at 7 years exerted indirect effects on disordered eating and depressive symptoms at 14 years via later higher BMI (at 11 years), depressive symptoms (at 11 years), and advanced breast development (at 13 years), with body dissatisfaction (at 11 years) serving as an additional mediator for disordered eating. Among boys, BMI at 7 years had indirect effects on disordered eating at 14 years via later BMI and body image (at 11 years), whereas only childhood depressive symptoms at 11 years had an effect on later depressive symptoms (at 14 years). These findings elucidate pathways to the development of eating pathology and depressive symptoms in adolescence, and in particular reveal a novel risk factor of advanced breast development for girls. The present study suggests childhood targets for preventative interventions, including higher BMI, body dissatisfaction, and depressive symptoms, in addition to advanced breast development for girls.
